# Mechanisms of Hearing Loss after Blast Injury to the Ear

**DOI:** 10.1371/journal.pone.0067618

**Published:** 2013-07-01

**Authors:** Sung-Il Cho, Simon S. Gao, Anping Xia, Rosalie Wang, Felipe T. Salles, Patrick D. Raphael, Homer Abaya, Jacqueline Wachtel, Jongmin Baek, David Jacobs, Matthew N. Rasband, John S. Oghalai

**Affiliations:** 1 Department of Otolaryngology–Head and Neck Surgery, Stanford University, Stanford, California, United States of America; 2 Department of Otolaryngology–Head and Neck Surgery, Chosun University, Gwangju, South Korea; 3 Department of Bioengineering, Rice University, Houston, Texas, United States of America; 4 Department of Computer Science, Stanford University, Stanford, California, United States of America; 5 Department of Neuroscience, Baylor College of Medicine, Houston, Texas, United States of America; Pomona College, United States of America

## Abstract

Given the frequent use of improvised explosive devices (IEDs) around the world, the study of traumatic blast injuries is of increasing interest. The ear is the most common organ affected by blast injury because it is the body’s most sensitive pressure transducer. We fabricated a blast chamber to re-create blast profiles similar to that of IEDs and used it to develop a reproducible mouse model to study blast-induced hearing loss. The tympanic membrane was perforated in all mice after blast exposure and found to heal spontaneously. Micro-computed tomography demonstrated no evidence for middle ear or otic capsule injuries; however, the healed tympanic membrane was thickened. Auditory brainstem response and distortion product otoacoustic emission threshold shifts were found to be correlated with blast intensity. As well, these threshold shifts were larger than those found in control mice that underwent surgical perforation of their tympanic membranes, indicating cochlear trauma. Histological studies one week and three months after the blast demonstrated no disruption or damage to the intra-cochlear membranes. However, there was loss of outer hair cells (OHCs) within the basal turn of the cochlea and decreased spiral ganglion neurons (SGNs) and afferent nerve synapses. Using our mouse model that recapitulates human IED exposure, our results identify that the mechanisms underlying blast-induced hearing loss does not include gross membranous rupture as is commonly believed. Instead, there is both OHC and SGN loss that produce auditory dysfunction.

## Introduction

Improvised explosive devices (IEDs; commonly used as roadside bombs) are a common cause of combat injury in the wars in Afghanistan and Iraq. They are also often used as weapons of terror, inflicting severe injuries on non-combatants around the world, including within the United States. When an explosive detonates, high pressure gasses are released that expand away from the point of detonation. This compresses the surrounding air and produces both a *blast wave* and a *blast wind* that propagate away from the explosion in a spherical pattern [1]. The high energy forces associated with these components of a blast can produce devastating trauma upon soldiers and civilians in its vicinity. Damage to the ear is the most common consequence of a blast exposure [2]. Among Veterans with service-connected disabilities, tinnitus is the most prevalent and hearing loss is the second-most prevalent condition [3]. Thus besides the obvious disabilities resulting from damage to the ear, there are significant long-term health care costs for society.

There are four different mechanisms of bodily damage after blast exposure. Primary blast injury is caused by the direct effect of the high pressure wave upon the tissue. Secondary blast injuries occur when the blast wind propels shell fragments or debris into the tissue. Tertiary blast injury is when the blast wind knocks down or blows the individual into a solid object. Quaternary blast injuries include all other effects, such as post-traumatic stress disorder or burns [4]. Primary blast injury is most noticeable where density changes markedly, such as tissue-air junctions [5]. Therefore, damage to the ear is a primary blast injury. Other organs that are particularly sensitive to primary blast injury include the lung and abdomen [6], [7].

While lethal blast pressures for humans are roughly 414–552 kPa, there is a 50% rate of tympanic membrane perforation with blast pressures of only 104 kPa [5]. Damage to the ear is an incredibly prevalent condition and over 60% of wounded-in-action service members have eardrum injuries, tinnitus, and/or hearing loss [8], [9]. As well, hearing loss is found post-deployment in 28% of all military personnel [10]. Some damage to the ear can be identified by clinical examination, such as perforations of the tympanic membrane. Damage to the middle ear ossicles are detectable by computed tomography. If spontaneous healing does not happen, surgical repair gives excellent results. Thus, blast injuries to the tympanic membrane and middle ear, while common, are not predominant causes of long-term disability.

The most devastating effect of blast injury to the ear is permanent hearing loss, due to cochlear trauma. However, our knowledge of the specific effects of blast damage on the human cochlea is minimal. The objective of this study was to develop a model that would allow us to study blast trauma similar to that produced by IEDs upon the mouse cochlea. We then used this model to measure changes in auditory thresholds as well as to quantify the tissue and cellular damage within the cochlea.

## Materials and Methods

### Animals

Mice were used in accordance with our experimental protocol that was approved by Institutional Animal Care and Use Committee at Stanford University (APLAC-23785). We used 5 to 6 week old male or female wild-type CBA/CaJ mice for all experiments. These mice have stable auditory thresholds from post-natal day 21 through 12 months of age [11]. Mice were anesthetized using ketamine and xylazine, and all efforts were made to minimize suffering.

### Blast Chamber

We custom-built a blast chamber to deliver blast waves to mice (Fig. S1). This system is pressurized with compressed air, which when released, produces a single compression wave that travels a down a PVC tube (length: 272 cm, outer diameter: 11.5 cm, wall thickness: 0.6 cm). As it travels down the tube, a shock front develops, creating a blast wave by the time it reaches the mouse. The reservoir within the blast chamber was pressurized using a standard air compressor (Model C2002-WK, Porter-Cable, Towson, MD) connected by a flexible hose. A gauge in the reservoir permitted us to fill it to the desired pressure level and thus produce blast waves of different strengths.

The blast wave profile impacting each mouse was measured for every experiment using a high-speed pressure transducer (Model 102B16, PCB piezotronics, Depew, NY) that was positioned just below the mouse, 11 cm from the end of the tube. We collected the pressure data dynamically using a signal conditioner (Model 482A21, PCB piezotronics) and digital oscilloscope (Model TDS2014B, Tektronix, Beaverton, OR). Our chamber could generate peak pressures of up to 186 kPa, corresponding to a sound intensity of 199 dB SPL (sound pressure level) at the position of the mouse.

After induction of anesthesia, the mouse was securely positioned at the end of the tube with its head facing directly into the force of the blast wave. In order to minimize trauma to the mouse other than primary blast injury on the ear, we protected its body by wrapping it in a sheath of fiberglass screen mesh (Insect Screening, Phifer Inc., Tuscaloosa, AL) and tape (Gorilla Glue Inc, Cincinnati, OH). We made small openings in the sheath to allow the auricles to protrude out and thus be exposed to the blast wave. We found this approach worked well because there was an immediate mortality rate of <20% at the highest blast pressures. This was a dramatic improvement over the immediate mortality rate of >50% for other positioning techniques we initially tried, which included placing the mice loosely in a wire-mesh cage, holding them sideways in plastic mesh, or securing them in a soft cloth sack. Occasionally, a mouse would die the first night after the blast, but this was rare (<5%). Thus, we believe this positioning strategy, as well as the aerodynamic nature of the sheath, helped to divert the blast wave and blast wind around the body of the mouse.

### High-speed Video Recording

We used a custom high-speed video recording system to image the force of the blast upon the mouse. The system consisted of a digital camera (A504kc, Basler, Ahrensburg, Germany) connected to a 50 mm lens (Canon, Tokyo, Japan) interfaced to a framegrabber (Karbon-CL, Bitflow, Inc., Woburn, MA) via dual-channel Camera Link protocol. The camera supports region-of-interest readouts, and we configured the camera to stream raw data from the first 512 rows of the sensor (or equivalently, frames at 1280×512 resolution at 8 bits per pixel) at 1000 fps. As a result, the camera could stream frames at 4.88 Gb/s, which was just under the maximum bandwidth of dual-channel Camera Link, specified as 5.44 Gb/s.

A custom computer was also built and programmed to store the data at high speed. The images received by the framegrabber were buffered in RAM via direct memory access, and were written onto a solid-state drive through the PCI-e x4 bus in chunks of 40 frames in order to maximize write throughput. The sustained write throughput of the solid-state drive in this scheme, at roughly 3 Gb/s, was lower than the throughput of incoming data. However, the system was programmed to be able to capture frames for roughly a one minute period of time by accumulating the extra data in memory. In post-processing, a demosaicing algorithm was used to reconstruct full color images. The stored frames were then color-corrected and encoded into a single complete video file.

### Microscopic and Endoscopic Exams of the Tympanic Membrane

The tympanic membranes of the mice were examined with either a dissecting microscope (OPMI1, Zeiss, Germany) or a rigid endoscope (2 mm straight endoscope, Stryker, Kalamazoo, MI).

### Micro-computed Tomography (CT) Exam

Radiographic imaging was performed using a micro-CT scanner (Imtek/Siemens MicroCAT II/SPECT system, Simens Medical Solutions, Malvern, PA). The mice were placed in the prone position for the study. The resolution of micro-CT was 40 µm in the X, Y, and Z dimensions. Images were analyzed in MicroView software (Version 2.1.2, GE Healthcare), Velocity (Version 6.0.1, Perkin-Elmer, Waltham, MA), and ImageJ (Version 1.46i, National Institutes of Health).

### Auditory Brainstem Responses (ABRs) and Distortion Product Otoacoustic Emissions (DPOAEs)

ABRs and DPOAEs were measured as previously described (Xia et al. 2007). We used custom hardware based around a data acquisition board (PCIe-6251, National Instruments, Austin, TX) driven by software we wrote in MATLAB. Briefly, the ABR potentials were measured from needle electrodes positioned at the bottom of the tympanic bulla and at the vertex of the head, with a ground electrode placed in the rear leg. The sound intensity level was raised in 10 dB steps from 10 to 80 dB SPL and the sound frequency was varied between 4 to 64 kHz. At each sound level, 260 responses were sampled and averaged. The maximum peak-to-peak value of the ABR (typically wave III of the signal) was measured and the threshold at each frequency was calculated to be when this value was five standard deviations above the noise floor. If an ABR response was not detected at 80 dB SPL, we arbitrarily set the threshold to be 80 dB SPL for averaging purposes.

DPOAEs were measured by a probe tip microphone in the external auditory canal. The sound stimuli for eliciting DPOAEs were two 1 second sine-wave tones of differing frequencies (F2 = 1.2×F1). We varied the range of F2 from 4 to 46 kHz. The two tones were of equal intensities and stepped from 20 to 80 dB SPL in 10 dB increments. The amplitude of the cubic distortion product was measured at 2*F1–F2. The threshold at each frequency was calculated to be when the DPOAE was >5 dB SPL and two standard deviations above the noise floor. If a DPOAE was not detected at 80 dB SPL, we arbitrarily set the threshold to be 80 dB SPL for averaging purposes.

### Plastic-embedded Histology to View Cochlear Cross-sections

After removing the temporal bones and opening the tympanic bullae, the stapes were removed and the cochleae were bathed in a mixture of 2.5% glutaraldehyde and 1.5% paraformaldehyde in 0.1 M phosphate buffered water (PB) at 4°C overnight. We did not mechanically perfuse the fluid chambers of the cochleae in order to minimize mechanical trauma unrelated to the blast. The specimens were rinsed with dH_2_O three times for 5 minutes and placed in 1% osmium tetroxide for 45 minutes. The cochleae were rinsed again with dH_2_O three times for 5 minutes and decalcified in 0.12 M EDTA in 0.1 M PB with 1% glutaraldehyde, with the pH adjusted to 7.0. The specimens were placed on a gentle tilting-type shaker at room temperature for three days and the EDTA solution was changed every day. Once decalcified, the cochleae were rinsed twice for 15 minutes in dH_2_O before they were dehydrated in 50% ethanol then 70% ethanol, for 15 minutes each time. This was followed by two changes of 95% ethanol for 15 minutes and four changes in 100% ethanol before the final dehydration step of 30 minutes in propylene oxide (PO).

The cochleae were gradually incorporated into Araldite with medium hardness (all percentages are by volume: 46.6% Araldite 502- #10900, 39.6% DDSA- #13710, 12.1% DBP- #13100, and 1.6% DMP-30-#13600, Electron Microscopy Sciences, Hatfield, PA) through 1∶1 mix of Araldite to PO for two hours and then 2∶1 mix of Araldite to PO overnight at room temperature. Finally, the cochleae were immersed in Araldite for 2 hours in a vacuum at room temperature, orientated to the desired position in a coffin mold filled with degassed Araldite, and placed in 60°C to harden for at least three days. The specimens were sectioned serially at either 10 or 20 µm thickness (RM2255, Leica, Buffalo Grove, IL). Sections were stained with 1∶10 dilution of Epoxy Stain (#14950, EMS, Hatfield, PA) with dH_2_O for two minutes and then washed in tap water. The dry sections were embedded with ClearMount (MMC0126, American MasterTech) and the coverslips were sealed with nail polish. The slides were viewed on an upright microscope (Axio Scope.A1, Zeiss, Germany) and images were taken using a color camera (AxioCam MRc, Zeiss, Germany).

### Whole-mount Preparations to View the Hair Cell Epithelium

Excised cochleae were fixed in 4% paraformaldehyde at room temperature for 1 hour and then immersed in 0.5 M EDTA for 5 hours. They were then rinsed three times (5 minutes per rinse) in PBS containing 0.1% Triton-X100 (PBST) at pH 7.4. The organs of Corti were then dissected free from the cochleae under a microscope.

In some preparations, phalloidin labeling (Liu et al. 2011) was performed to image actin, a major component of the hair cell stereocilia and cuticular plate. The cochleae were permeabilized with 0.1% Triton X-100 in PBS and simultaneously stained with 1∶200 Alexa 488 Phallodin for 15 minutes at room temperature.

In other preparations, immunolabeling was performed by first blocking the organs of Corti with 4% donkey serum (017-000-121, Jackson Immuno Research Laboratories, West Grove, PA) in PBST for 1 hour at room temperature and then incubating with the primary antibody at 4°C overnight. Specimens were washed three times with PBST and then incubated with the secondary antibody at room temperature for 1 hour. We used antibodies to label either OHCs (prestin) or synaptic ribbons (CtBP2) and a neuronal marker, neural class III beta-tubulin (TUJ1). Both labeling protocols were combined with a general hair cell marker (myosin VIIa). For OHC labeling, the primary antibodies were goat anti-prestin N-20 (1∶500; SC-22692, Santa Cruz Biotechnology, Santa Cruz, CA) and rabbit anti-myosin VIIa (1∶200; Proteus Biosciences Inc., Ramona, CA). For synaptic ribbon labeling, the primary antibodies were goat anti-CtBP2 (1∶200; SC-5967, Santa Cruz Biotechnology), mouse anti-Tuj1 (1∶200; MO15013, Meuromics, Edina, MN) and rabbit anti-myosin VIIa (1∶200; Proteus Biosciences Inc.). The secondary antibodies we used were Alexa Fluor 488 donkey anti-rabbit, Alexa Fluor 546 donkey anti-mouse, and Alexa Fluor 633 donkey anti-goat (all used at a dilution of 1∶500; Invitrogen, Grand Island, NY).

After washing with PBST again, the specimens were mounted with Prolong Antifade (Invitrogen). Images were acquired with a Zeiss LSM5 Pascal system using 20X/0.5 EC Plan-NEOFLUAR, 63X/1.4 Oil Plan-Apochromat, and 100X/1.4 Oil Plan-Apochromat objectives. When indicated, the complete length of the cochlea was carefully reconstructed by overlapping the common cells at the edges of the individual images in Photoshop (Version 11.0, Adobe System Inc, San Jose, CA). Cytocochleograms were then made by counting all inner and outer hair cells and clustering them into 20 different locations relative to their distance from the base of the cochlea, using 5.72 mm as the average length of a CBA/CaJ mouse [12].

### Immunolabeling with Sectioned Tissue

Cochleae were fixed in 4% paraformaldehyde at 4°C overnight before cryoprotection in a sucrose gradient (10%–30%) and embedding in OCT for cryo-sectioning. Serial sections with thickness of 7 µm were washed three times before blocking for one hour in normal donkey serum at room temperature. They were then incubated with the primary antibody in phosphate buffered saline containing 0.1% Triton-X100 (PBST) overnight at 4°C. Sections were washed three times with phosphate buffered saline (PBS) and then incubated with the respective secondary antibodies at room temperature for one hour. They were washed three times, 10 minutes each time, in PBST before mounting with Fluoroshield with DAPI (Sigma, St. Louis, MO). The primary antibodies were rabbit anti-neurofilament 200 (1∶200), mouse anti-GFAP (1∶500) (N206A/8, UC Davis/NIH funded Neuromab monoclonal antibody resource), and rabbit anti-IBA1 (1∶200) (polyclonal antibody, Wako Chemicals). The secondary antibodies were donkey anti-mouse Alexa Fluor 488 and donkey anti-rabbit Alexa Fluor 546 (1∶500; Invitrogen). Images were taken using the epi-fluorescence function of a LSM 5 Exciter upright microscope (AxioImager, Zeiss, Germany).

### Statistical Analyses

Statistical analyses were performed using Excel (Microsoft, Seattle, WA) and SPSS (Systat Software). Comparisons of averaged data were performed using the one-way ANOVA (for three categories) and the Student’s t-test (for two categories). ABR and DPOAE thresholds were compared using two-way ANOVA using frequency and test date as the two independent variables. P values <0.05 were considered statistically significant. All p-values are provided in the figure legends. Error bars are the SEM.

## Results

### Blast Wave Characteristics

**Figure 1:**
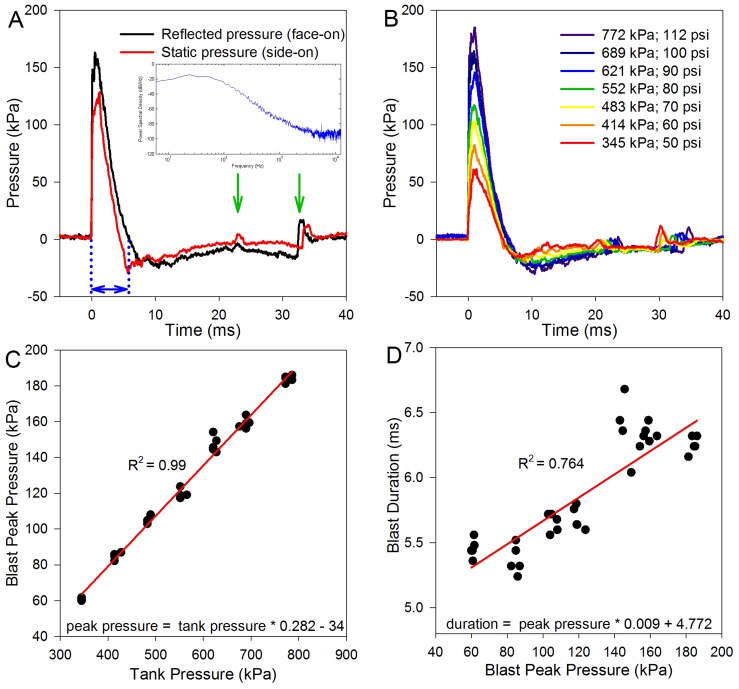
Blast wave characteristics, measured without a mouse in the tube. (**A**) The reflected and static pressures were measured to describe the blast wave profile. The stagnation pressure was measured with the pressure sensor as shown in Figure 1D (facing the oncoming blast wave). The static pressure was measured with the pressure sensor turned 90°, so that the sensing surface was facing vertically (side-on to the blast wave). Note the rapid onset of the blast wave at 0 ms, the blast wind peak about 2–3 ms later, and then the under-pressure from ∼6–20 ms. These waveforms are characteristic of that seen with an explosive detonation. The arrows highlight reflected waves that occurred outside of the blast chamber. The inset is an average power spectral density analysis of the stagnation pressure from five blast waves. (**B**) Varying the pressure in the reservoir chamber changed the blast wave profile. (**C**) There was a linear relationship between the reservoir chamber (tank) pressure and the peak blast pressure. (**D**) Larger magnitude blasts produced slightly longer blast durations, consistent with a longer blast wind.

We first characterized our blast chamber using two different methods to measure the pressure of the blast wave at the same location (**Fig. 1A**) [13]. We measured the stagnation pressure by positioning the pressure sensor (face-on to the blast wave as shown in **Fig. S1D**), whereas the static pressure was measured by having the pressure sensor protrude through a hole in the bottom wall of the tube (i.e. side-on to the blast wave). The static pressure measurement does not assess the dynamic pressure associated with particle motion, whereas the stagnation pressure is the sum of the static and dynamic pressures. Both pressure measurements were made the same distance from the end of the tube.

Both measurements demonstrated that there was an initial overpressure peak followed by a negative pressure phase. These data revealed that the blast wave conformed to the theoretical ideal for a blast wave as given by a Friedlander function [14]. There was an immediate rise at the onset of the blast that corresponds with the blast wave (0 ms) and the blast wind could be seen as the slower rise to the peak blast pressure (2 ms). The pressure then dropped below the baseline as the blast wave and wind propagated past the sensor and then slowly recovered. There were two small perturbations in the pressure signal (arrows) that originated from reflections of the blast wave. The duration of the blast was the time from the onset of the blast to the zero-crossing point (blue arrows). For all remaining experiments, only the stagnation pressure measurements were performed. A power spectral density analysis of five blasts was performed and averaged (**inset,** **Fig. 1A**). This demonstrated that most of the blast energy was below 1 kHz, although there was energy out to 12.5 kHz (the maximum frequency we could analyze based on sampling rate) and an energy peak at 5 kHz.

The minimum reservoir pressure necessary to move the components inside the chamber and produce a blast was roughly 345 kPa (50 psi). The maximum reservoir pressure we arbitrarily decided to limit to 793 kPa (115 psi). We then plotted representative blast wave profiles versus time at different reservoir pressures (**Fig. 1B, C**). This demonstrated that higher reservoir pressures produced higher blast pressures. As well, while the onset profile of the blast wave demonstrated a step response at higher reservoir pressures, there was an onset rise-time associated with the lower reservoir pressures (compare the first 1 ms of the bottom and top tracings in Fig. 1B). This indicates that the shock front had not developed as well when using lower pressures compared to higher pressures. Nevertheless, higher peak blast pressures were associated with slightly longer blast times (**Fig. 1D**), consistent with the production of higher magnitude blast waves and blast winds.

When the peak blast pressures were converted from Pascals to decibels of sound pressure level (SPL) to estimate the intensity of the sound, the range was from 189–199 dB SPL. At the average sea-level air pressure of 101.325 kPa (our blast chamber in Palo Alto is fired at an altitude of roughly 100 ft above sea level), the maximum sound intensity level is calculated to be 194 dB SPL. Thus, blast pressures above this level presumably resulted in supersonic propagation velocities. Altogether, these findings indicate that our blast chamber was able to repeatedly produce blast waves reasonably similar to those produced by high explosives. However, the non-spherical propagation of the overpressure and the lack of a damping system at the end of the blast tube led to a larger-than-typical blast wind [13]. Importantly, this indicates that the mice that were exposed to the blast in these experiments had an experience similar to that of humans exposed to an IED. For reference, common IED blast exposures that cause trauma have peak pressures that range from 10–200 kPa and positive phase durations of 4–10 ms [15], [16], [17].

To demonstrate the blast procedure, we recorded video of test blasts on the roof of our building. Then, to visually assess the impact of the blast wave upon the mice, we recorded some actual experiments using a custom-built high-speed camera (**Video S1**). We matched the time sequence of the video images with those of the blast wave profile. As seen, the mouse was supported within the center of the blast tube and only the ears and tail were visible. The mouse together with the plastic mesh that surrounds it moved back-and-forth but not side-to-side because of the positioning system. Therefore, it did not hit the walls of the tube and injuries to organs other than the ears were minimized.

### Auditory Brainstem Responses (ABRs) and Distortion Product Otoacoustic Emissions (DPOAEs)

**Figure 2:**
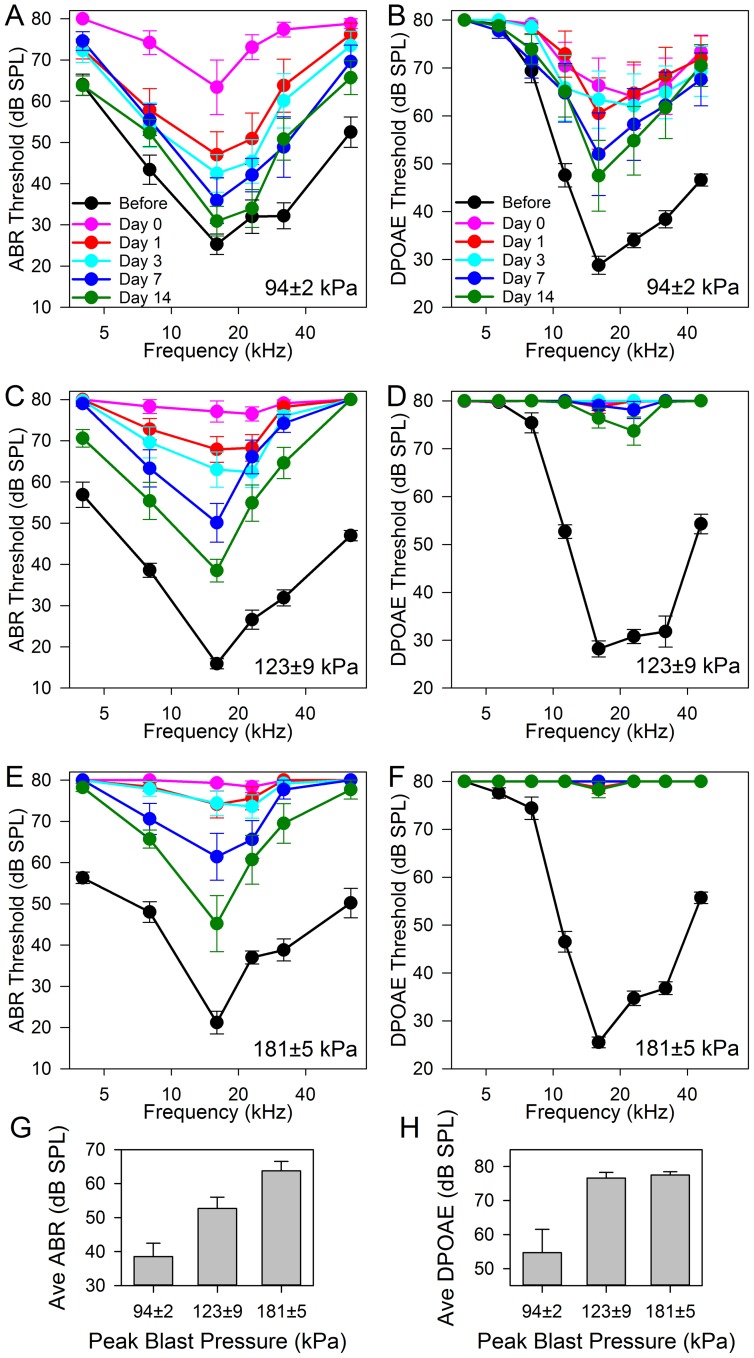
ABR and DPOAE thresholds across the frequency spectrum for cohorts of mice exposed to different blast peak pressures. The blast peak pressure is given in the lower right of each plot. (**A,B**) The lowest blast pressure cohort had a nearly complete recovery of ABR thresholds and a partial recovery of DPOAE thresholds within two weeks. However there were still statistically significant differences between the ABR and DPOAE thresholds before the blast compared to 14 days after blast (two-way ANOVA, p<0.001 for both sets of curves). (**C,D**) By 14 days, the middle blast pressure cohort had less recovery of ABR thresholds and almost no recovery of DPOAE thresholds (two-way ANOVA, p<0.001). (**E,F**) The highest blast pressure cohort had ABR and DPOAE thresholds that were even larger, and by 14 days remained higher than the thresholds before the blast (two-way ANOVA, p<0.001). (G,H) The average of the ABR and DPOAE thresholds at 16, 23, and 32 kHz (the most sensitive frequencies) two weeks after the blast. Higher peak blast pressures led to higher thresholds for both ABR and DPOAE averages (one-way ANOVA, p<0.001).

We then assessed the effect of the blast exposure on auditory thresholds. We exposed three cohorts of five mice (10 ears each) to different blast pressures distributed over the peak blast pressure range of the chamber (94±2 kPa, 123±9 kPa, and 181±5 kPa). We then repeatedly measured ABR and DPOAE thresholds in these cohorts for 14 days (**Fig. 2**).

In the first cohort (94±2 kPa), ABR and DPOAE thresholds were substantially elevated immediately following the blast exposure (Day 0). These elevations were over the entire frequency spectrum. Over the subsequent two weeks, there was a gradual partial recovery of the thresholds. While the ABR thresholds nearly recovered completely in the lower frequencies (<30 kHz), there were still elevations in the higher frequencies (>30 kHz). In contrast, the DPOAE thresholds demonstrated much larger elevations over the entire frequency spectrum. The other two cohorts (123±9 and 181±5 kPa) had ABR threshold shifts that demonstrated larger initial threshold elevations and less recovery. However, DPOAE thresholds showed little-to-no recovery over the frequency spectrum.

We then compared average thresholds at the most sensitive frequencies between cohorts 14 days after the blast. The average of the ABR thresholds at 16, 23, and 32 kHz demonstrated elevations that correlated with the peak blast pressure (**Fig. 2G**). A similar effect was seen with the average of the DPOAE thresholds at 16, 23, and 32 kHz (**Fig. 2H**). The difference in the average DPOAE thresholds in the 123 and 181 kPa cohorts may be small because the 80 dB SPL maximum stimulus intensities used for these experiments limited our ability to detect differences at extreme levels.

### The Tympanic Membrane and Ossicular Chain

**Figure 3:**
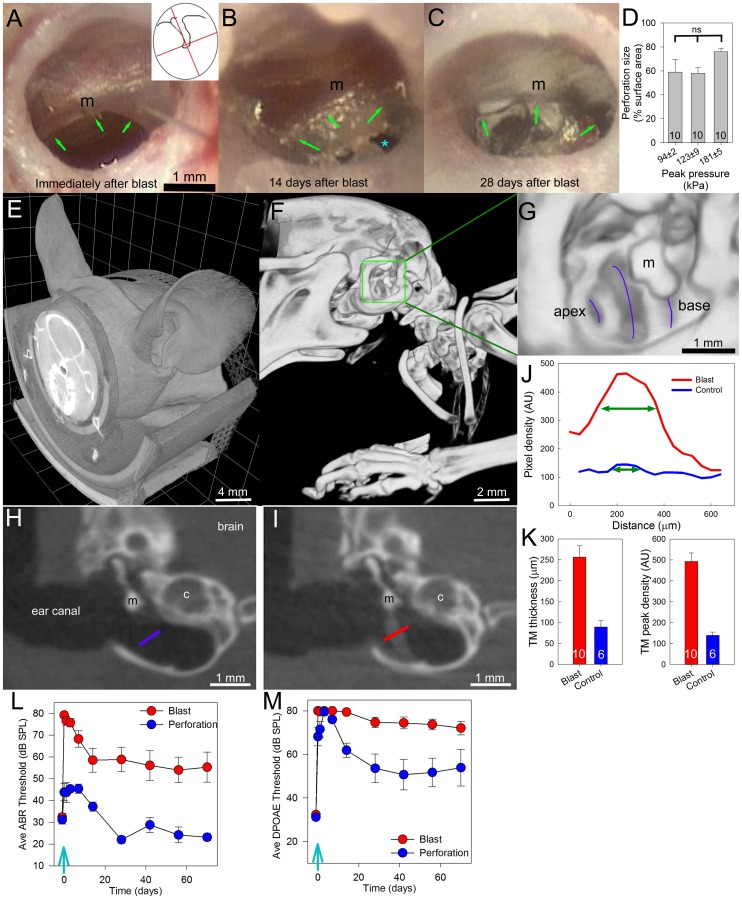
Perforation of the tympanic membrane after blast exposure. Each image is from a different mouse. (**A**) Immediately following a blast, perforations were always seen as in this representative example (*green arrows*). The malleus (*m*) is identified. To estimate the size of the perforation, the tympanic membrane was considered to have four quadrants (*inset*) each containing 25%. In this example, the perforation was estimated to be 40% of the surface area of the tympanic membrane. (**B**) By 14 days after the blast, it was typical for the perforations to be partially healed. The original edges of the perforation (*green arrows*) and a portion of the perforation that had not healed (*blue asterisk*) can be seen. (**C**) By 28 days after the blast, most of the perforation had healed. The original edges of the perforation in this representative example are highlighted (*green arrows*). (**D**) There was no difference in the size of the perforation between different peak blast pressures (n = 10 for each group; one-way ANOVA, p>0.1). (**E**) Micro-CT of the mouse demonstrates that most of the head was scanned. (**F**) To assess for skull fractures, the image contrast and brightness were adjusted to remove the soft tissues. (**G**) The temporal bone was enlarged, demonstrating the turns of the cochlea (*purple lines*). The cochlear apex and base are identified. Malleus (*m*). No fractures of the skull or otic capsule bone were ever noted (n = 10). (**H**) A coronal cross-section through a representative control (i.e. age-matched) mouse temporal bone. The blue line traverses the tympanic membrane. Malleus (*m*); cochlea (*c*). (**I**) A representative image from a mouse three months after blast exposure demonstrates a thickening of the inferior portion of the tympanic membrane. (**J**) The signal density profile of the tympanic membranes shown in (H) and (I). The thickness was calculated as the width at half-maximum (*green lines*). (**K**) The tympanic membrane (*TM*) thickness and peak density were higher in blast-exposed mice compared to controls (Student's non-paired t-test, p<0.001 for each measure). (**L,M**) Long-term changes in ABR and DPOAE thresholds. One cohort of ten mice was exposed to the highest blast pressure (*blast*) and another cohort of mice underwent surgical perforation of their tympanic membranes (*perforation*). Auditory thresholds were repeatedly measured for 70 days in both cohorts. The average of the ABR and DPOAE thresholds at 16, 23, and 32 kHz in both cohorts stabilized after 28 days. However, thresholds remained higher in the blast-exposed mice (non-paired T-test, p<0.001 for each measure), consistent with permanent cochlear damage.

Perforations of the tympanic membrane were seen in all mice after the blast exposure (**Fig. 3A–D**). The perforations always occurred within the inferior aspect of the tympanic membrane. The size of every perforation was estimated visually by the same observer using a well-validated technique used in the clinic to assess patients with tympanic membrane perforations [18], [19], [20]. With this technique, the perforation is compared against a model where the tympanic membrane is divided into four equal sections defined by two lines, one along and one perpendicular to the malleus (**Fig. 3A**-***inset***). Thus, each quadrant represents 25% of the surface area of the tympanic membrane. All estimates of perforation size were made to the nearest 5^th^ percentile. These data indicated that the size of the perforation ranged from 20% to 100% immediately after the blast. Larger blast pressures did not make larger perforations (ANOVA, p = 0.136).

We then took the cohort of mice exposed to the highest blast pressures (181±5 kPa; n = 10 ears from five mice) and followed them for three months to assess the spontaneous ability of the tympanic membrane to heal. Initially after the blast, the average tympanic membrane was 24% ±3%intact (range 20–50%). One month after the blast, the average tympanic membrane was 93% ±2%intact indicating that substantial healing of the perforations had already occurred (three were completely healed and seven had partially healed). By two months, this rate was 95% ±2% (five had completely healed and five had only partially healed). By three months, the average tympanic membrane was 96% ±2% intact (five had completely healed and five had only partially healed; range 80–100%).

After three months, we used micro-CT to study the otic capsule bone, ossicular chain, and tympanic membrane in these mice (**Fig. 3E–K**). We compared these results to those of unexposed control mice of the same age and background (n = 6 ears from three mice). We sequentially studied each section through every ear looking for injuries, fractures, and scars. We did not find any evidence of fracture or scarring of the otic capsule bone or of the ossicular chain. As well, there was no evidence of ossicular dislocation. Lastly, we did not note any layering of fluid in the middle ear space, as might be expected with leakage of perilymph through a cochlear fistula or a rupture of the oval window or round window. Thus, blast exposure produced no detectable damage to the bony structures of the middle and inner ear.

The tympanic membrane, however, had clear evidence of healed perforations (**Fig. 3H–K**). We quantified the scarring within the tympanic membrane by plotting the pixel intensity along a line orthogonal to the mid-inferior tympanic membrane (the region of the perforations). The maximum pixel intensity along this line represented the peak density of the tympanic membrane and the width at half-maximum intensity was used as the thickness of the tympanic membrane. The peak density of the tympanic membrane was higher in blast-exposed mice compared to controls (493.1±40.3 vs. 123.0±26.7 A.U.). As well, the thickness of the tympanic membrane was greater in blast-exposed mice than in controls (243±16 vs. 99±11 µm). Thus, even though the tympanic membranes healed after blast injury, they were denser and thicker than normal.

### Long-term Threshold Shifts

We followed a cohort of 5 mice (10 ears) for 70 days after exposure to blast pressures of 181±5 kPa. Their ABR and DPOAE thresholds were measured repeatedly during this time frame. In order to estimate the effect of tympanic membrane trauma, we also followed a cohort of mice that were not exposed to blasts. Instead, on day 0, we visualized the left tympanic membrane under the microscope and manually created a perforation of ∼50–60% using a pick. Thus, we could assess the impact of an isolated tympanic membrane perforation on ABR and DPOAE recovery in the mouse. The cohort consisted of 3 mice (6 ears).

The thresholds in blast-exposed mice (**Fig. 3L,M**) demonstrated partial recovery within the first 28 days. There were no substantive changes in ABR or DPOAE thresholds after day 28. Similar timelines were seen for ABR and DPOAE threshold changes in mice that had their tympanic membranes perforated. However, there were obvious differences in the degree of hearing recovery. Compared to baseline, the ABR threshold elevations were ∼25 dB in blast-exposed mice whereas there was no change in mice with surgically-perforated tympanic membranes. As well, the DPOAE threshold elevations were ∼40 dB in blast-exposed mice whereas they were ∼20 dB in mice with surgically-perforated tympanic membranes.

These threshold differences were not due to differences in tympanic membrane healing. Seventy days after the injury, blast-exposed mice had tympanic membranes that were 96% ±2% intact while the surgically-perforated tympanic membranes were 99% ±1% intact (p>0.2). Thus, we conclude that the damage resulting from the blast exposure produced not only injury to the tympanic membrane, but also injury to the cochlea or auditory nerve, i.e. sensorineural hearing loss.

### Cochlear Histology

**Figure 4:**
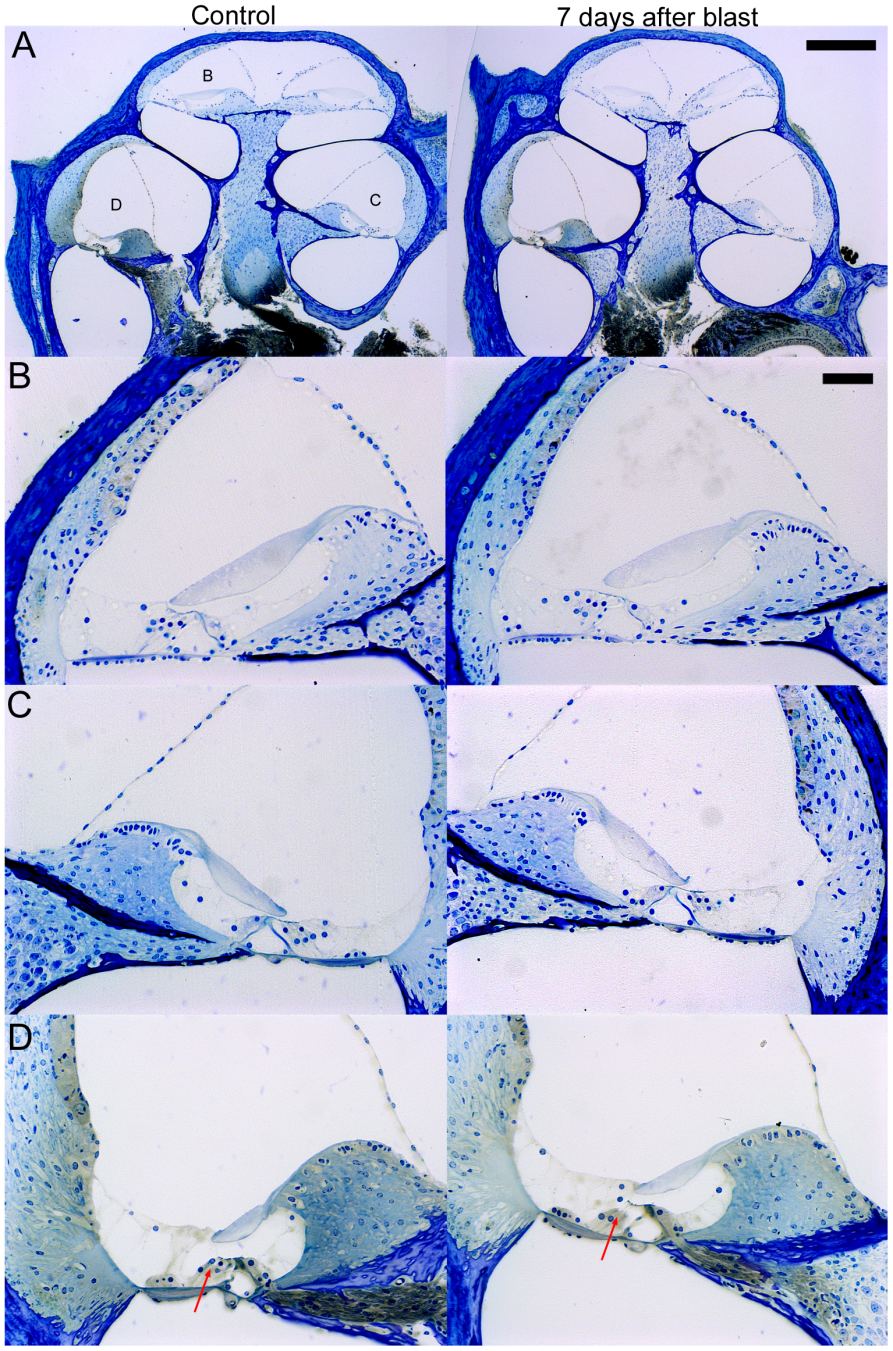
Representative plastic-embedded cochlear sections of age-matched controls and seven days after blast and. The sections were 10 µm thick. (**A**) The complete cochlear cross-sections are shown with labels indicating the areas that are enlarged. (**B,C,D**) Enlargements of the apical turn (**B**), the upper basal turn (**C**), and the lower basal turn (**D**). There was no evidence for obvious gross disruption of the intracochlear soft tissues. However, there was apparent loss of OHCs in the basal region of the cochlea as assessed by loss of their dark-stained nuclei (compare arrows in D). Scale bars: A-250 µm, B-50 µm.

We examined plastic-embedded, cochlear cross-sections for evidence of trauma in both blast-exposed and age-matched control mice. We studied 10 sequential sections from each of 8 blast exposed and 7 control mice. The sections were taken within the center of the cochlea and contained the mid-modiolar region (**Fig. 4**). The peak blast pressures all were within the range of the two highest pressure categories, where the physiology indicated the presence of sensorineural hearing loss (range: 120–185 kPa). Four blast and four control specimens were collected three months after the blast and an additional four blast and three control specimens were collected seven days after the blast.

We did not see any qualitative differences in the mice sacrificed three months after the blast compared to those sacrificed seven days after the blast. Specifically, we assessed each turn for ruptures in Reissner’s membrane or the basilar membrane, elevation of the tectorial membrane from the hair cell epithelium, and changes in the staining intensity and cellular morphology of the stria vascularis and lateral cochlear wall. Thus, there was no evidence for gross cochlear trauma. However, one common finding that we did note in all blast-exposed mice was a lack of OHCs in the lower basal region, as noted by the lack of the row of three darkly-stained nuclei above the Deiter’s cell nuclei (**Fig. 4D**).

### Hair Cell Studies

**Figure 5:**
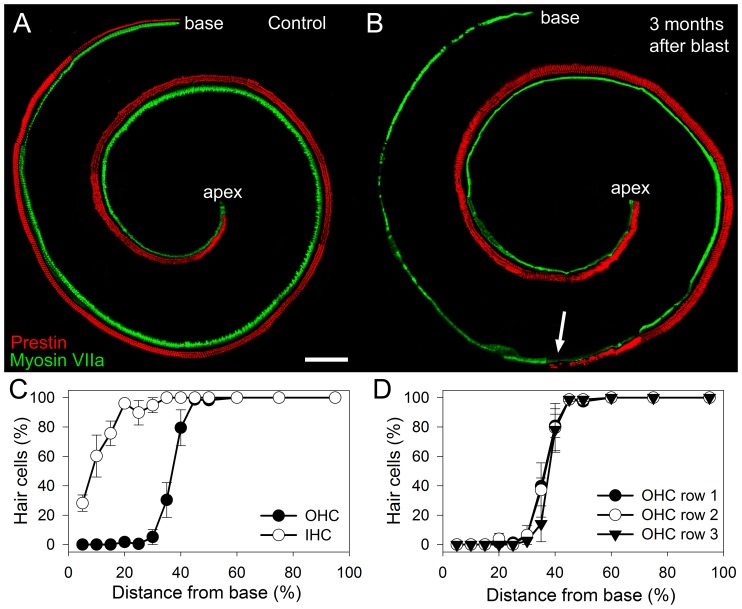
Representative whole mount preparations of the cochlear epithelium immunolabeled for prestin and myosin VIIa. OHCs are red and IHCs are green. (**A**) An age-matched control mouse demonstrates the full complement of OHCs and IHCs. Scale bar 100 µm. (**B**) Three months after blast-exposure, substantial OHC loss was found within the basal turn. While some IHCs were missing, most were present. The transition zone roughly 30% up from the base of the cochlea (*arrow*) marked the point at which some OHCs were able to survive the blast trauma. (**C**) Cytocochleograms were performed for quantification in mice three months after blast. (**D**) There were no differences in the patterns of OHC loss between the three rows in mice after blast.

Immunolabeling for prestin, an OHC-specific protein, and myosin VIIa, a protein found in both OHCs and inner hair cells (IHCs) was performed on whole mount preparations of the cochlear epithelium (**Fig. 5A,B**). This was performed in mice three months after exposure to blast pressures of 181±5 kPa and in age-matched controls. Images were collected using a confocal microscope at multiple positions, which were then connected together to study the length of the cochlea. At each position, the focus was adjusted to image either the OHCs or the IHCs, and the filter set changed to collect either the green or the red channel. Thus, OHCs were red and IHCs were green.

There was an orderly alignment of the three rows of OHCs and single row of IHCs. However, missing hair cells were easily identifiable as a lack of fluorescence. We counted the hair cells and created cytocochleograms to assess the relationship between hair cell loss and cochlear location (**Fig. 5C,D**). Hair cell loss was highest at the base and declined at more apical locations. In fact, there was no hair cell loss in the apical half of the cochlea. As well, the loss of OHCs was remarkably larger than the loss of IHCs. There was no difference in the degree of OHC loss between the three rows.

**Figure 6:**
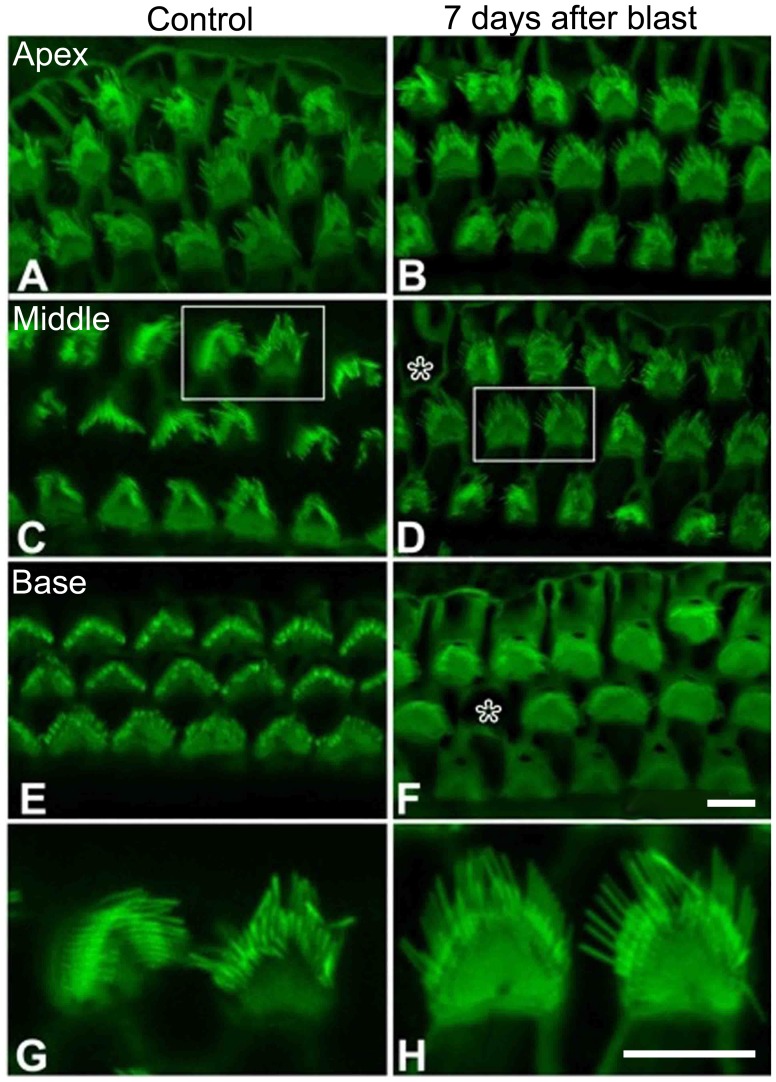
Confocal imaging of phalloidin-stained cochleae. Residual OHCs seven days after blast exposure do not have gross disturbances of their stereociliary bundles. Asterisks indicate missing OHCs. (**A,B**) The apex of the cochlea. (**C,D**) The middle of the cochlea. (**E, F**) The base of the cochlea. While blast-exposed mice did not have any residual OHCs present within the far base of their cochlea, shown here is a cluster of residual OHCs at the transition zone (arrow in Fig. 5B). (**G, H**) Enlargements of the OHCs indicated by the white boxes in parts C&D. In all images, the normal stereociliary bundle morphology is seen. The scale bar is 8 µm.

We also studied cochlea from mice seven days after blast exposure of 181±5 kPa and from age-matched controls using phalloidin labeling (**Fig. 6**). For these studies, high-resolution confocal imaging was performed to assess for stereocilia disarray and hair cell loss. While it is well-recognized that some artifact is normal with whole-mount preparations in the adult mouse cochlea, we did not see any unusual stereocilia morphology in the blast-exposed mice. All observed hair cell stereociliary bundles retained the typical staircase arrangement, the typical V-shape at the apical surface, and the presence of tapered cilia. This was found even in the transition zone of blast-exposed mice, the point at which some OHCs were able to survive the blast (as delineated by the arrow in Fig. 5 B).

### Spiral Ganglion Neuron Studies

**Figure 7:**
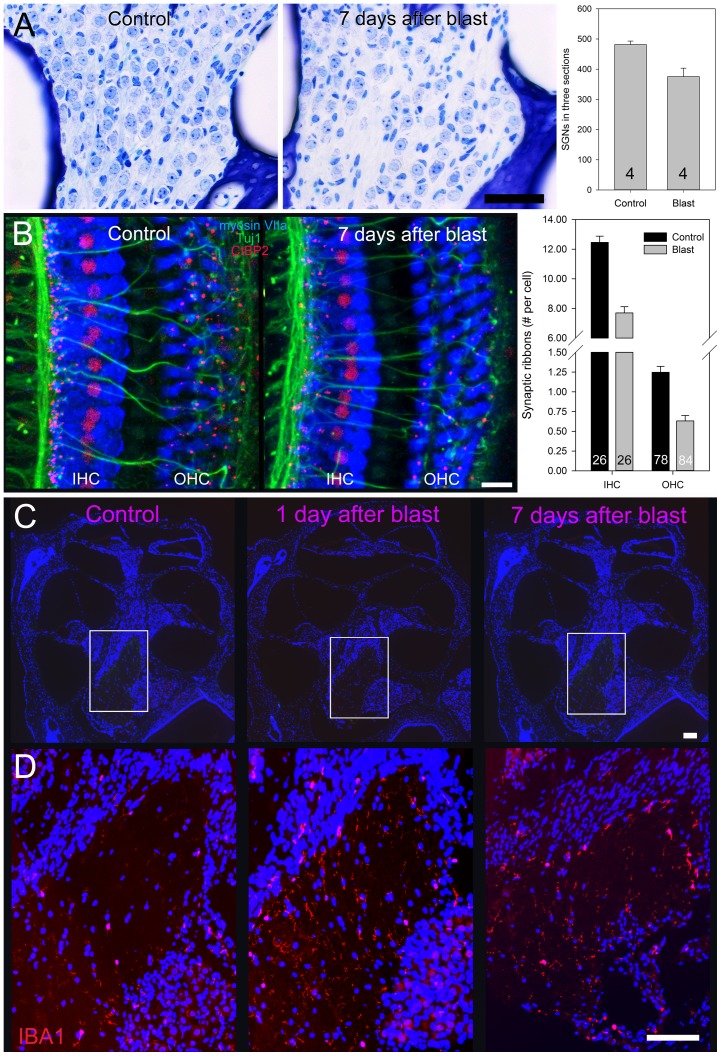
Spiral ganglion neurons. (**A**) Plastic embedded sections of control mice and mice seven days after blast exposure. There was a significant reduction of spiral ganglion neurons (*SGNs*) in blast-exposed mice compared to controls (non-paired Student's t-test, p = 0.013). SGNs were identified by their larger nuclei and prominent nucleoli. (**B**) Summed confocal Z-stack images of the apical turn of the cochlea in control and mice seven days after blast exposure. The number of synaptic ribbons (*red punctate labeling*) under the IHCs and the OHCs was reduced after blast exposure (non-paired Student's t-test, p<0.0001 for both). (**C**) Representative paraffin embedded cochlear cross-sections stained with DAPI from a control mouse, a mouse one day after blast exposure, and a mouse seven days after blast exposure. The boxes highlight the modiolus, which was expanded for the immunolabeling studies in (D). (**D**) IBA1 expression was stronger in mice one and seven days after blast exposure. Scale bars: A-50 µm, B-20 µm, C-200 µm, D-200 µm.

During our review of the plastic-embedded sections, we noted that there were less spiral ganglion neurons (SGNs) in blast-exposed mice (**Fig. 7A**). We quantified this effect by counting their cell bodies within the modiolus and Rosenthal’s canal of four mice three months after blast exposure (181±5 kPa) and four age-matched control mice. For each cochlea, we summed the total number of SGNs from three mid-modiolar sections. There was a significant reduction of SGNs in blast-exposed mice compared to controls (376±28 vs. 482±12, respectively).

To further characterize the injury, we labeled afferent synapses using antibodies to CtBP2, a structure associated with synaptic ribbons, which are found on the presynaptic (hair cell) side of the synapse. Antibodies to Tuj1 were also used to label the nerve fibers. While >95% of afferent synapses are associated with IHCs (to type I spiral ganglion neurons), there are some afferent synapses associated with OHCs as well (to type II spiral ganglion neurons) [21]. We focused our analysis on the apical and middle regions of the cochlea, as this is where there was no evidence of hair cell loss. We found a high density of punctate labeling along the IHCs and scattered punctate labeling along the OHCs in control mice (**Fig. 7B**). However, seven days after blast exposure (181±5 kPa), there was an obvious decrease in the density of labeling under both IHCs and OHCs.

We performed Z-stacks, reconstructed them in 3D, and then carefully counted the number of fluorescent puncta associated with each IHC and OHC from two control and two blast exposed mice (two z-stacks were collected from each cochlea in both the 8 and 12 kHz regions). We found that there was a significant decrease in the number of synaptic ribbons associated with IHCs after blast exposure (7.69±0.43 vs. 12.46±0.42 per IHC). Similarly, there was a reduction in the number of synaptic ribbons associated with OHCs between the two cohorts (0.63±0.07 vs. 1.25±0.07 per OHC). This indicates that even though there was no hair cell loss in the apical region of the cochlea after blast exposure, there was loss of spiral ganglion innervation to that region. Because this large degree of loss was found in the apical regions of the cochlea and affected both IHCs and OHCs, the amount of loss we noted was more extensive than what has been shown in noise-exposed mice [22]. Nevertheless, the overall findings are consistent with what has been previously published, and indicates that the blast exposure not only leads to loss of afferent nerve fibers, but also produces changes in the intracellular molecular morphology of residual hair cells.

Finally, we performed immunolabeling of paraffin-embedded cochlear cross-sections taken from control mice, mice 1 day after blast exposure (181±5 kPa), and mice 7 days after blast exposure (**Fig. 7C**). We immunolabeled for ionized calcium binding adaptor molecule 1 (Iba1), a protein that is upregulated within activated microglia and has been used as a marker of inflammation [23] (**Fig. 7D**). Compared to controls, there was a slight increase in Iba1 expression within the modiolus one day and seven days after blast exposure. Together, these data support the concept that loss of SGNs associated with blast trauma produced inflammation within the cochlear modiolus.

## Discussion

The popular conception of blast injury to the cochlea is that there is traumatic disruption of the intracochlear soft tissues, such as the basilar membrane, Reissner’s membrane, the reticular lamina, and/or the separation of the supporting cells from the basilar membrane. Our findings in a mouse model exposed to a blast wave and wind equal to or greater than that of a typical IED injury sustained by a soldier suggest that this pathology is not typical. In contrast, we found that the gross cochlear anatomy was unaffected whereas the predominant pathologic findings were OHC loss in the basal 40% of the cochlea and SGN loss. The hair cells that survived had normal stereociliary bundle morphology, however they had loss of synaptic ribbons.

Our assessment of the consequences of blast trauma upon cochlear function was contaminated by hearing loss due to tympanic membrane perforations. Even though the perforations healed, auditory thresholds remained elevated due to this confounding factor. We attempted to estimate the impact of healed tympanic membrane perforations on auditory function by comparing the blast-exposed mice to mice that had surgically-perforated tympanic membranes. While the surgically-perforated mice had full recovery of their ABR thresholds, their DPOAE thresholds remained elevated. This result is consistent with a persistent conductive hearing loss, which has a greater impact on DPOAE than ABR thresholds [24]. The change in auditory thresholds in the mice due to cochlear trauma alone can be estimated by subtracting the ABR and DPOAE threshold shifts in the surgically-perforated mice from those of the blast-exposed mice. Therefore, we estimate that the sensorineural hearing loss caused by the blast-induced cochlear trauma produced ∼25 dB and ∼20 dB elevations in ABR and DPOAE thresholds, respectively. However, because DPOAE thresholds were often not measurable in blast-exposed mice at our equipment limits, it is possible that we are underestimating the DPOAE threshold shift. In any case, these magnitudes of threshold shifts are similar to those found in transgenic mice with malformations where the tectorial membrane contacts some, but not all, of the OHCs [25].

If the tectorial membrane had separated from the epithelium and was not contacting any of the OHCs, ABR threshold elevations would be ∼30 dB higher than what we found [26]. As well, the tectorial membrane did not appear elevated off of the epithelium in our plastic-embedded sections, which is somewhat surprising given how commonly this histological artifact is found even with normal, unexposed mice. Thus, we conclude that tectorial membrane-OHC separation after blast exposure is unlikely, and it is reasonable to assume that the tectorial membrane is interacting with the OHCs that remain after a blast injury to support partial function of the cochlear amplifier. We did not note any rupture of the epithelium as has been seen 24 hours after noise exposure [27]. However, this trauma heals to form an undifferentiated epithelium within 1–2 weeks, the time period where we did our histological studies. Our finding of complete loss of outer hair cells in the base of the cochlea is certainly consistent with what would be expected after a disruption of the reticular lamina has scarred and healed.

Given the involvement of the U.S. military personnel in conflicts around the world and the prevalence of IEDs, the study of traumatic blast injuries is important. Combat body armor provides soldiers with considerable protection against penetrating ballistic injury, yet it does not protect against the effects of blast overpressure on the ear. In fact, the ear is typically the first organ affected with a primary blast injury because it is the body’s most sensitive pressure transducer [28], [29], [30]. Tympanic membrane perforation is the most common combat-related ear injury [31]. Indeed, tympanic membrane perforation has been used as a convenient biomarker for blast injuries to other organs that may be more difficult to diagnose, such as traumatic brain injury [32], [33], [34]. The fact that all of our blast-exposed mice sustained tympanic membrane perforations supports the relevance of this model not only for the study of hearing loss, but also potentially for the study of other organ systems.

An important concept in establishing an animal model of blast-induced hearing loss is to minimize injuries other than primary blast injuries to the ear. The most common complications include primary blast injury to the lungs, which causes pulmonary contusions, and tertiary blast injury, where the animal’s body moves and hits a cage or the wall of the tube. Both complications often produce severe trauma and lead to animal death. We overcame these potentially confounding issues by developing the technique of suspending the mouse in the middle of the tube and using plastic mesh to shield the body from much of the blast force. Similar techniques have been used by others in rats, where the mortality was significantly higher when the unprotected rat body was exposed to blast, compared to head-directed, body-protected blast impacts of similar magnitude [35]. As well, traumatic brain injury, memory loss, and damage to the axon initial segment were found [36].

Another aspect important for this project was that the blast chamber needed to appropriately simulate the blast faced by a person exposed to an IED. We used a compressed air mechanism, in which the force of the blast was directed down a length of PVC tubing. This approach is similar to that used by many other groups [13], [37], [38]. A major benefit of this approach is that it focuses the blast energy into a small volume so that actual explosives are not needed to produce the target peak blast pressures experienced in a free space environment [39]. Thus, experiments are much safer and more feasible.

However, one downside to this approach is that the blast wind can be larger than it would be in free space with the same blast wave pressure. This is because as the blast wave leaves the open end of the tube, it dissipates and pulls more air out of the tube (which generates the blast wind). We attempted to minimize this effect by placing the mouse as far in the tube as feasible, while still being able to access it for multiple experiments. As well, we described the magnitude of this effect by measuring both the static and stagnation pressure waveforms at the location of the mouse (see **Fig. S1A**). Both waveforms had similar shapes, and the static pressure was about 80% that of the stagnation pressure. The peak of the blast wind was slightly higher than that of the blast wave whereas the blast wave peak is higher than the blast wind peak with TNT explosions in free space [13]. This indicates that our blast chamber produces blasts that accurately simulate IED explosions with the caveat that the blast wind is slightly larger than is typical, which may increase the damage sustained by the ear. Other than this, the blast patterns generated by our chamber are reasonably consistent with an explosion in free space. Obviously, it is impossible to perfectly re-create in the lab what a person in proximity to an IED explosion experiences because IEDs produce varying degrees of blast pressures and blast winds depending upon their orientation, shielding, and the position of the affected person.

Our finding of OHC loss in mice in the cochlear base is similar to other reports describing blast-induced hearing loss in rats [37] and after noise exposure in mice [40]. The SGN loss we noted also is similar to that seen after noise exposure in mice [27], [41], [42], [43]. These findings are consistent with the fact that humans exposed to a blast typically have partial, rather than complete, hearing loss. We found that mice had similar auditory threshold shifts and subsequent recovery responses after blast exposure to previously published data in the cat and the chinchilla after impulse noise exposure [44], [45], [46], [47], [48]. However, we did not find evidence of substantial gross cochlear trauma, such as tearing or rupture of the sensory cells from their supporting cell attachments on the basilar membrane, trauma of the reticular lamina, and particulate debris within the scala fluid chambers as others have seen [49], [50], [51], [52], [53]. These studies were performed in chinchilla and pigs, and it is possible that these species, either because of their larger size or for other reasons, are more susceptible to trauma than the mouse. As well, these reports applied multiple impulse noise exposures, rather than a single blast exposure as we did. Lastly, it should be noted that these reports were predominantly histological surveys looking for evidence of blast trauma. As the adult mammalian cochlea is notorious for being heavily influenced by artifact, it is conceivable that some of the reported findings of trauma were in fact due to dissection trauma or tissue shrinkage during fixation, dehydration, and/or embedding.

We used genetically-identical CBA mice in this study. This is beneficial because it reduces variability between animals, improving the power of the study and the reliability of the results. Thus, animal usage is reduced. As well, the opportunity to use transgenic mice exists, in which certain genes may be able to be linked to higher or lower levels of cochlear trauma after blast. Thus, a genetic basis for blast-induced hearing loss might be identified. However, a downside of using only one mouse strain is that if its genetic background is unknowingly associated with an increased or decreased resistance to blast trauma, a finding that is important to humans may be either missed or exaggerated, respectively. We used CBA mice which are commonly used in studies of noise-induced hearing loss [54]. Although they have been shown to have increased noise susceptibility compared to C57Bl/6J mice at younger ages, they do not have the age-related hearing loss associated with this strain [55], [56]. As well, other strains of mice have been shown to have even more alterations in noise susceptibility [57]. Thus to summarize, a caveat of this study is that the genetic background and age of the mice we used may impact the pathology we found.

The power spectrum of the blast energy created by our chamber is within the audible range of humans. We performed these experiments personally and, even though we use high-quality hearing protection, we can vouch for the potential danger of an accidental exposure. We certainly would expect damage to the human cochlea from a blast similar to what our chamber creates. However the main question is what happens when a human is exposed to a real blast from an IED? Until a cochlear imaging technique suitable for use in living patients becomes available, the actual damage sustained by a human cochlea will remain unknown. Optical coherence tomography is one emerging technology that may provide an answer to this question in the future [58], [59]. While this study demonstrates OHC and SGN loss by one week after the blast, presumably the cell death occurs soon after the blast trauma. By understanding the pathophysiology of the mechanisms underlying this degenerative pathway, it may be possible to develop the necessary surgical techniques and/or drugs to reduce the degree of permanent cochlear damage. Further studies during this critical time period following blast exposure are underway to elucidate these mechanisms.

## Supporting Information

Figure S1 **The blast chamber.** (**A**) A schematic diagram of the blast chamber. The toggle clamp is pushed in and the air compressor is used to fill the reservoir chamber to the desired level, as measured by the pressure gauge. (**B**) Firing a blast is initiated by pulling the toggle clamp back (*top*). The high pressure within the reservoir chamber can then enter the blast tube (*middle*). Finally, the blast wave propagates down the blast tube (*bottom*). (**C**) Picture of the blast chamber. The orientation is the same as in (A). The oscilloscope is used to record the blast wave profile as measured by the pressure sensor. (**D**) The end of the blast tube contained the mouse in its protective sheath and the pressure sensor. (**E**) The blast chamber, toggle clamp, and pressure gauge.
(TIF)

Video S1 **The blast chamber and blast exposure protocol.**
(MP4)
