# Supplementary figures and images for: Mechanisms of Hearing Loss after Blast Injury to the Ear
Source: PLoS One. 2013 Jul 1;8(7):e67618. doi: 10.1371/journal.pone.0067618 (PMC3698122; doi:10.1371/journal.pone.0067618)

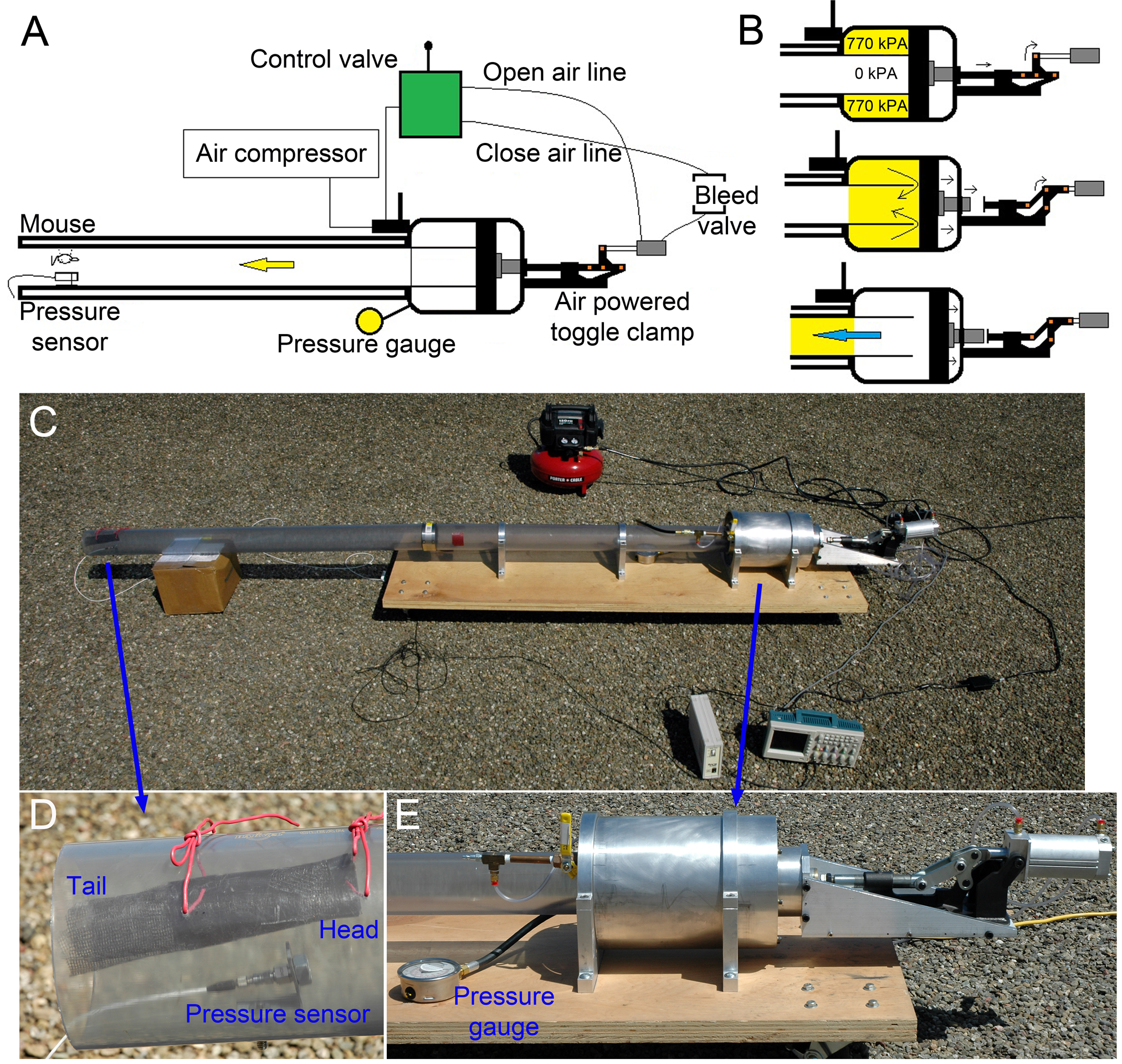

Supplement: Figure S1 — The blast chamber. (A) A schematic diagram of the blast chamber. The toggle clamp is pushed in and the air compressor is used to fill the reservoir chamber to the desired level, as measured by the pressure gauge. (B) Firing a blast is initiated by pulling the toggle clamp back (top). The high pressure within the reservoir chamber can then enter the blast tube (middle). Finally, the blast wave propagates down the blast tube (bottom). (C) Picture of the blast chamber. The orientation is the same as in (A). The oscilloscope is used to record the blast wave profile as measured by the pressure sensor. (D) The end of the blast tube contained the mouse in its protective sheath and the pressure sensor. (E) The blast chamber, toggle clamp, and pressure gauge. (TIF) [file pone.0067618.s001.tif]
